# No Impaired Glucose Tolerance in Primary Insomnia Patients with Normal Results of Polysomnography

**DOI:** 10.3389/fneur.2017.00303

**Published:** 2017-06-27

**Authors:** Johanna Tschepp, Christoph J. Lauer, Johanna Wilde-Frenz, Thomas Pollmächer

**Affiliations:** ^1^Klinikum Ingolstadt, Centre of Mental Health, Ingolstadt, Germany

**Keywords:** sleep disturbances, glucose metabolism, insomnia, glucose tolerance test, normal sleep

## Abstract

**Background:**

According to recent studies, sleep restriction and disruption both have a prominent negative influence on glucose metabolism. This could also be shown in sleep disorders, such as sleep apnea and the restless legs syndrome. However, similar studies regarding insomnia have not been that consistent, yet. Moreover, most previous studies did not include objective polysomnography (PSG) data.

**Methods:**

Patients with primary insomnia (*N* = 17) and healthy controls (*N* = 15) were investigated using psychometric tests such as the Epworth Sleepiness Scale and the Pittsburgh sleep quality index (PSQI). Two nights of full PSG were performed in all subjects, and after the first PSG night subjects underwent a standard oral glucose tolerance test (OGTT). PSG-, arousal-, and glucose metabolism-parameters were compared between groups.

**Results:**

Patients with insomnia were, as expected, sleepier than healthy controls and showed higher PSQI values. All PSG parameters, however, including parameters related to nocturnal arousals, did not differ between groups. Moreover, OGGT results and all other parameters of glucose tolerance were not different between insomniac patients and healthy controls.

**Conclusion:**

Our findings suggest that glucose tolerance is not impaired in patients with chronic insomnia and normal PSG-findings. Therefore, impaired glucose metabolism and diabetes related to insomnia in earlier studies might be restricted to those patients who have objectively disturbed sleep.

## Introduction

Recent studies clearly show that sleep deprivation and impairments of sleep continuity compromise glucose metabolism. In healthy humans this is true for sleep restriction for several hours to some days (1, 2) and for experimental sleep disruption, e.g., by acoustic stimulation (3). All these challenges acutely impair glucose tolerance mainly by compromising insulin sensitivity (1, 4). Although the underlying mechanisms are not clear yet, increased sympathetic tone and heightened hormonal stress system activity in sleep disturbed subjects are likely to play an important role (5, 6).

These experimental data suggest that clinical sleep disorders might compromise glucose tolerance, too and, in addition, increase the risk of these patients to develop diabetes (7, 8). Indeed, numerous studies have reported impaired glucose tolerance (IGT) in sleep disorders such as the restless legs syndrome (RLS) (6) and obstructive sleep apnea syndrome (OSAS) (9, 10). A recent study of our group (6) found, in addition to an IGT, a positive correlation between the number of arousals related to periodic leg movements (in RLS) or to apneic events (in OSAS) and morning fasting glucose, further supporting the idea that sleep disturbed by arousals has particularly negative effects on glucose metabolism.

In the same study patients with primary insomnia did not show any abnormality of glucose metabolism as compared to healthy controls. This is somewhat surprising, because earlier studies suggest impaired glucose metabolism in insomnia patients as well (11, 12). However, in all of these studies, the sleep of insomnia patients were not investigated objectively by polysomnography (PSG), and in our earlier study PSG was not available for the healthy controls. According to all relevant diagnostic systems, i.e., ICD-10 (13), DSM-IV (14), the recently released DSM-V (15), and the ICSD-2 (16), primary insomnia is defined by subjective criteria only. Thus, it is not surprising that reported PSG findings vary widely (17–19), ranging from almost unremarkable sleep amount and measures of sleep continuity to severely disturbed sleep. This variation in sleep disturbance could also explain the variation in findings regarding glucose metabolism as already suggested by Vgontzas and colleagues (20). However, there is no study so far to compare sleep and glucose metabolism of primary insomnia patients to healthy controls simultaneously.

Therefore, we studied nocturnal sleep and glucose tolerance in patients with chronic primary insomnia, carefully selected for the absence of any other sleep or psychiatric disorders, with age-, gender-, and body-mass-index-matched controls.

## Materials and Methods

### Ethics Statement

This study was carried out in accordance with the recommendations of the Bavarian Medical Council, Munich, Germany with written informed consent from all subjects. All subjects gave written informed consent in accordance with the Declaration of Helsinki. The protocol was approved by the Bavarian Medical Council, Munich, Germany (commission number: 09052).

### Participants

The participants were recruited by advertisements in the local press and in our outpatient sleep disorders department. We investigated 32 subjects between the ages of 25 and 65. Seventeen suffered from chronic primary insomnia, according to DSM-IV (21). Fifteen subjects were healthy body-mass-index-, age-, and gender-matched controls.

Prior to admission to the study, subjects underwent a detailed check-up. This check-up consisted of a medical and psychiatric interview, a physical examination, and anthropometric measurements. Several blood tests were performed, including a complete hemogram, prothrombin clotting time, activated partial thromboplastin time, fibrinogen, aspartate aminotransferase (AST/GOT) and alanine aminotransferase (ALT/GPT), LDH, lipase, cholesterol, HDL-cholesterol, LDL-cholesterol, albumin, transferrin, ferritin, iron, CRP, TSH, T3, T4, and cortisol levels, potassium, chloride, sodium, calcium, magnesium, phosphate, and urine drug monitoring. All participants had normal results of a medical and neurological examination. Subjects with sleep disorders (except insomnia in the experimental group, of course), other medical or psychiatric disorders were excluded. Shift-workers, pregnant women, and people who had traveled across multiple time zones within the last 3 months prior to the study were not allowed to take part in the study, either. All participants were off any medication affecting sleep for at least 2 weeks. To make sure that the participants had a regular sleep–wake-schedule, they wore a wrist activity monitor (Cambridge Neurotechnology, Cambridge, UK) for 8 days. An ambulant apnea screening (Weinmann, Somnocheck, Hamburg, Germany) was performed to exclude subjects with sleep-related breathing disorders.

### Procedures and Measurements

After entering the study, the subjects completed several questionnaires including the Epworth Sleepiness Scale (ESS) (22) to assess daytime sleepiness and the Pittsburgh Sleep Quality Index (PSQI) (23), measuring the sleep quality. The Beck Depression Inventory (BDI) (24) and the Hamilton Depression Scale (HAMD) (25) were used to assess depressive symptoms, and anxiety symptoms were measured using the Hamilton Anxiety Scale (HAMA) (26).

Two nights of standard PSG were performed. The first night served for the adaption to the sleep laboratory conditions. Only data from the second night were used for our present analysis. The PSG was recorded from 11:00 p.m. to 6:00 a.m. using a Schwarzer Harmonie system (Version 6.1, Munich, Germany). PSG included the electroencephalographic (EEG) recordings (C4-A1 and C3-A2), the electrooculogram (EOG), an electromyogram (EMG) (submental and from the right and left tibialis anterior muscle), an electrocardiogram (ECG), recordings of the thoracic and abdominal movements, nasal airflow, pulse oximetry, and video monitoring. Sleep stages were defined by EEG, EOG, and EMG according to the criteria of the American Academy of Sleep Medicine (27). Scoring was done by experienced scores blind to diagnostic status of the subjects. For each night, sleep onset (SOL, with reference to lights off), time of sleep in stage 1 (N1), stage 2 (N2), slow-wave sleep (SWS/N3), and rapid eye movement (REM) sleep were measured. Time in bed, total sleep time, wake time after sleep onset (WASO), sleep efficiency (SEI), sleep latencies in SWS and REM sleep, periodic leg movements during sleep-index (PLMS-index), and the apnea–hypopnea index were also calculated. Arousals were scored according to the American Sleep Disorders Association report on EEG arousals (28). We differentiated between two types of arousals: cortical and movement arousals. Movement arousals were defined by an additional rise in chin EMG amplitude and/or a rise in leg EMG activity. Then, the arousal-index, the cortical-arousal-index, and the movement-arousal-index were computed.

After the first night in the sleep lab—after an overnight fast—the participants underwent a 2-h oral glucose tolerance test (OGTT) at 8:00 a.m. A fasting sample (including HbA1c measurement) was taken at 0 min. After an oral standard load of 75 g glucose blood samples to measure the blood glucose level were taken at 30, 60, and 120 min. Glucose was measured by using the glucose oxidase method (Synchrone DXC 800 1 + 2, Beckmann Coulter, USA) with an inter-assay coefficient 1.0–2.2% (DXC 1) and 1.4–2.2% (DX2). The HbA1c was measured by using the colorimetric method, based on the assessment of the total hemoglobin concentration. The measurement of the A1c concentration was performed by the utilization of the turbidimetric-immunoinhibition-method (Synchrone DXC 800 1, Beckmann Coulter, USA) with an inter-assay coefficient of 2.9–3.2%.

According to the criteria of the American Diabetes Association (2003), normal glucose tolerance (NGT) was defined as a 2-h plasma glucose (2h-PG) concentration <140 mg/dl. IGT was defined as 2h-PG values ≥140 and <200 mg/dl. 2h-PG values above 200 mg/dl defined the diagnosis of diabetes. The combination of elevated HbA1c values (≥5.5%) and impaired fasting glucose values [fasting plasma glucose (FPG) ≥ 100 mg/dl] seems to be an additional risk factor for type 2 diabetes (29). Thus, the diabetes risk for the patients with insomnia and the healthy controls was calculated. Additionally, the area under the curve for glucose (AUCg) was determined by using the linear trapezoidal rule (30).

### Statistical Analysis

For statistical analysis, SPSS for Windows 19.0 (IBM Deutschland, Ehningen, Germany) was used. *T*-tests for independent samples were used to compare the mean values of the baseline parameters (age, sex, BMI, ESS, PSQI, BDI, HAMD, and HAMA), the sleep and the metabolic parameters (FPG, 2h-PG, and HbA1c) between the two groups. The chi-square test served to compare the incidence of an IGT and the diabetes risk between the two groups. Also, *p* < 0.05 was considered statistically significant.

## Results

Table 1 shows the baseline characteristics for all subjects. Both groups were matched regarding age, BMI, and sex. The patients with primary insomnia had a mean duration of the disorder of more than 10 years with a range from 1 to 34 years. None of the participants was affected by psychiatric disorders other than insomnia according to the results of a psychiatric examination. There were no significant differences in the BDI results between the patients with insomnia and the healthy controls. Insomnia patients had higher scores in the HAMD and HAMA, those scores, however, were far below the clinically relevant thresholds (see legend below Table 1). The subjects suffering from insomnia were sleepier than controls, as shown by higher values in the ESS. Patients with insomnia scored higher in the PSQI, depicting the impaired sleep quality compared to the healthy controls.

**Table 1:** Baseline parameters.

	Insomnia (*N* = 17)	Controls (*N* = 15)	*p*-values
Sex (male/female)	8/9	6/9	>0.05
Age	46.5 (11.1)	47.5 (10.2)	>0.05
Insomnia duration (in years)	10.8 (9.6) [1–34]	–	
Body-mass-index (kg/m^2^)	25.4 (2.5)	26.6 (4.6)	>0.05
Pittsburgh Sleep Quality Index	10.8 (3.0)	3.1 (1.6)	<0.001
Epworth Sleepiness Scale	8.8 (5.1)	4.8 (2.9)	<0.01
Beck Depression Inventory (BDI)	6.4 (3.6)	3.7 (5.2)	>0.05
Hamilton Depression Scale (HAMD)	3.0 (1.1)	0.3 (0.8)	<0.001
Hamilton Anxiety Scale (HAMA)	2.1 (0.7)	0.3 (0.9)	<0.001

*Data are mean (SD) [range]*.

*Cut-offs are as follows*:

*BDI: 0–10: no depression, 11–17: mild-moderate depression, ≥18: clinically relevant depression*.

*HAMD: 0–7: no depression, 8–13: mild depression, 14–18: moderate depression, 19–22: severe depression, ≥23: very severe depression*.

*HAMA: 0–13: no anxiety, 14–17: mild anxiety severity, 18–24: moderate anxiety severity, 25–30: severe anxiety*.

In Table 2 macro sleep parameters of the patients with insomnia and the controls can be seen. None of these sleep parameters differed significantly between both groups. Neither the SEI was reduced in the patients with insomnia nor was the wake time after sleep onset significantly higher compared with the controls.

**Table 2:** Macro structure of sleep.

	Insomnia (*N* = 17)	Controls (*N* = 15)	*p*-values
Time in bed (min)	436.9 (23.5)	432.5 (28.4)	>0.05
Sleep period Time (min)	423.4 (22.4)	415.1 (26.0)	>0.05
Total sleep time (min)	363.9 (29.2)	362.4 (22.3)	>0.05
Sleep efficiency (%)	86.1 (7.0)	87.6 (6.2)	>0.05
Sleep latency N1 (min)	10.2 (8.3)	8.6 (8.5)	>0.05
Sleep latency N2 (min)	12.0 (8.3)	11.0 (9.5)	>0.05
Wake time after sleep onset (min)	62.9 (30.7)	61.6 (27.9)	>0.05
Rapid eye movement sleep (min)	73.3 (23.6)	77.0 (21.6)	>0.05
N1 (min)	37.4 (14.8)	32.2 (10.7)	>0.05
N2 (min)	205.3 (23.3)	205.6 (33.5)	>0.05
N3 (min)	47.9 (21.2)	47.5 (22.1)	>0.05

*Data are mean (SD)*.

Table 3 displays the results of the micro structure of sleep. There were no significant differences in the arousal-index, the PLMS-index, and the other sleep parameters between both groups, either.

**Table 3:** Micro structure of sleep parameters.

	Insomnia (*N* = 17)	Controls (*N* = 15)	*p*-values
Apnea–hypopnea index	5.6 (6.8)	4.2 (2.6)	>0.05
PLMS-index per hour	9.8 (15.4)	5.8 (8.4)	>0.05
Total number of arousals	62. 9 (30.5)	65.7 (26.3)	>0.05
Number of cortical arousals	25.6 (14.3)	35.0 (27.1)	>0.05
Number of movement arousals	37.4 (28.8)	30.7 (16.8)	>0.05
Arousal-index per hour	10.5 (5.2)	10.8 (4.0)	>0.05

*Data are mean (SD)*.

**Figure 1:**
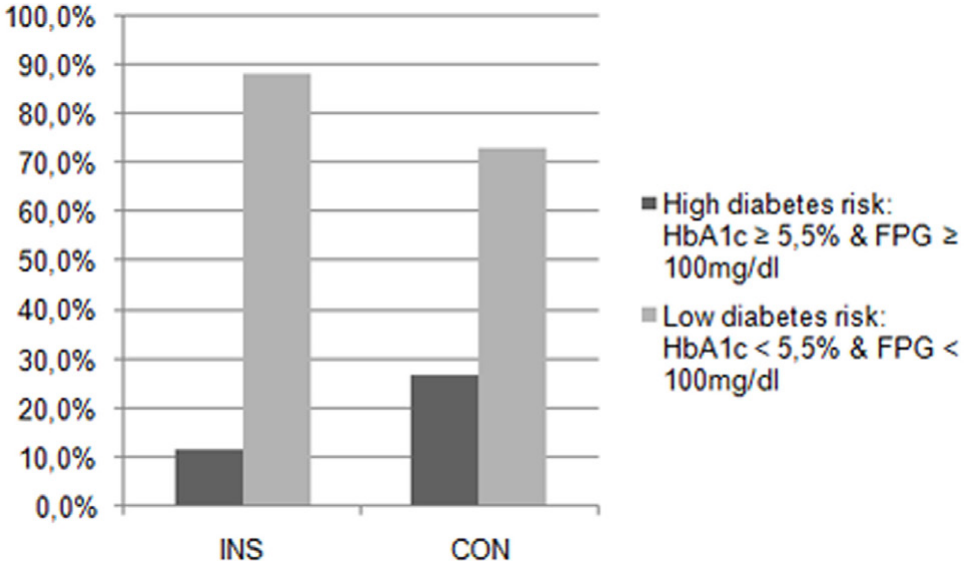
Diabetes risk in patients with insomnia (INS) and in the control group (CON). The patients with insomnia showed no significantly higher risk for diabetes than the controls.

Table 4 displays the parameters of glucose metabolism, particularly the HbA1c, the FPG, the blood glucose levels during the OGTT, and the AUCg in both groups. There were no significant differences in these parameters between the patients with insomnia and the controls. None of the participants suffered from diabetes according to the results of the OGTT. Table 5 and Figure 1 also indicate that the subjects suffering from insomnia were neither more likely to have an IGT nor was their risk to develop diabetes higher than in the control group. One hundred percent of the patients with insomnia and 80% of the controls had an NGT. Also, 20% of the controls showed an IGT.

**Table 4:** Glucose parameters.

	Insomnia (*N* = 17)	Controls (*N* = 15)	*p*-values
HbA1c (%)	5.3 (0.3)	5.4 (0.3)	>0.05
Fasting blood glucose (mg/dl)	97.6 (8.2)	96.3 (10.3)	>0.05
Blood glucose after 30 min (mg/dl)	138.7 (22.1)	156. 9 (30.3)	>0.05
Blood glucose after 60 min (mg/dl)	120.3 (20.8)	135.9 (49.9)	>0.05
Blood glucose after 120 min (mg/dl)	100.5 (14.8)	105.1 (28.7)	>0.05
Area under the curve for glucose (mg/dl)	14,043. 8 (1,319.7)	15,422.0 (3,710.5)	>0.05

*Data are mean (SD)*.

**Table 5:** Frequency of patients with normal glucose tolerance (NGT) and impaired glucose tolerance (IGT).

	Total	NGT	IGT
	*N*	*N* (%)	*N* (%)
Patients with insomnia	16	16 (100)	0 (0)
Controls	15	12 (80)	3 (20)

*Due to problems when taking the blood samples, it was only possible to examine 16 of the 17 patients with insomnia regarding NGT and IGT*.

*NGT: 2-h plasma glucose (2h-PG) < 140 mg/dl; IGT: 2h-PG ≥ 140 mg/dl*.

*χ^2^ (1) = 3.54*.

## Discussion

Considering the results reported above, it can be concluded that the patients with insomnia neither had any sign of objectively disturbed sleep nor did they have an increased number of arousals. Moreover, they did neither show an IGT nor an increased risk for diabetes compared to healthy controls. Although the patients with insomnia showed HAMD and HAMA scores significantly above controls, a clinically relevant degree of depression can be excluded, since (a) HAMD and HAMA scores were far below clinical thresholds and (b) patients did not fulfill diagnostic criteria for mood or anxiety disorders according to DSM-IV.

The findings suggest that even chronic insomnia without any objective alteration of the sleep-architecture does not influence the glucose metabolism in a negative way. This is congruent with the results of Keckeis et al. (6), who reported no IGT in patients with insomnia, either. However, Keckeis et al. found out that patients with OSAS and patients with RLS had an impaired glucose metabolism and a higher diabetes risk, which was attributable to arousals caused by apneas or leg movements, leading to an activation of the sympathetic nervous system. Correspondingly, as the patients with insomnia did not have an increased number of apneas, nor did they have an increased number of PLMS in our study, it is plausible that they did not show an increased number of arousals and thus there glucose metabolism was not altered in a negative way.

Inconsistent with our results is the study of Kachi et al. (11), who pointed out that there was a linear correlation between the HbA1c and difficulties maintaining sleep and early morning awakening. However, these findings are not comparable to the results of the present study, since Kachi et al. had not recorded any objective PSG-data.

A lot of studies give evidence for the fact, that disturbed sleep has a negative influence on glucose metabolism. Not only short sleep duration is associated with (7, 20, 31) but also a lack of slow-wave sleep is regarded as a risk factor for diabetes (32). However, these findings are not contradictory to the results of our study. In the present study, the patients with insomnia did not have an altered sleep-architecture in comparison to the controls. Their sleep length was not shortened, either. Therefore, insomnia without objectively disturbed sleep does not seem to lead to an impairment of the glucose metabolism. This is congruent with another study (33) that postulated that only insomnia combined with an objectively short sleep duration leads to a higher risk for diabetes.

The present study is limited by the fact that the number of subjects investigated was quite small. Hence, it is not possible to draw a statistically confirmative conclusion regarding equality between the two groups with respect to the prevalence of IGT. Statistical power calculated *ex post* amounts to 0.35 only, whereas values of 0.80 or above would be needed for robust conclusions. Conversely, 60 subjects per group would be necessary to achieve this power. The present study, being the first one relating glucose tolerance in insomnia to objective PSG results, might stimulate other researchers and their funding sources to address the issue in larger samples.

To conclude, further investigations with the focus on patients suffering from insomnia with objectively disturbed sleep might be useful. More studies including both metabolic and PSG-data are needed to increase our knowledge on the glucose metabolism of patients suffering from insomnia.

## Ethics Statement

This study was carried out in accordance with the recommendations of the Bavarian Medical Council, Munich, Germany, with written informed consent from all subjects. All subjects gave written informed consent in accordance with the Declaration of Helsinki. The protocol was approved by the Bavarian Medical Council, Munich, Germany (commission number: 09052).

## Author Contributions

JT conducted the study, analyzed data, and cowrote the paper; CL and JW-F co-analyzed data and cowrote the paper; TP designed the study, supervised the data analyses, and cowrote the paper.

## Conflict of Interest Statement

The authors declare that the research was conducted in the absence of any commercial or financial relationships that could be construed as a potential conflict of interest.
